# Anti-chaperone βA3/A1_102-117_ peptide interacting sites in human αB-crystallin

**Published:** 2008-03-26

**Authors:** Guruprasad Rao, Puttur Santhoshkumar, K. Krishna Sharma

**Affiliations:** 1 Department of Ophthalmology, University of Missouri-Columbia, Columbia, MO; 2 Department of Biochemistry, University of Missouri-Columbia, Columbia, MO

## Abstract

**Purpose:**

Our previous work identified 23 low molecular weight (<3.5 kDa) crystallin peptides in the urea-soluble fractions of normal young, normal aged, and aged cataract human lenses. We found that one of these crystallin fragments, βA3/A1_102–117_ peptide (SDAYHIERLMSFRPIC), that are present in aged and cataract lens, increased the scattering of light by β- and γ-crystallins and alcohol dehydrogenase (ADH) and also reduced the chaperone-like activity of αB-crystallin. The present study was performed to identify the interacting sites of the βA3/A1_102–117_ peptide in αB-crystallin.

**Methods:**

βA3/A1_102–117_ peptide was first derivatized with sulfo-succinimidyl-2-[6-(biotinamido)-2-{p-azidobenzamido}-hexanoamido] ethyl-1–3 dithio propionate (sulfo-SBED), a photoactivable, heterotrifunctional biotin-containing cross-linker. The biotin-derivatized peptide was then incubated with αB-crystallin at 37 °C for 2 h to allow complex formation followed by photolysis to facilitate the transfer of the biotin label from the peptide to αB-crystallin. Label transfer was confirmed by western blot, and the labeled αB-crystallin was digested with trypsin. Tryptic peptides from αB-crystallin carrying the biotin label were purified by avidin affinity chromatography, and βA3/A1_102–117_ peptide interacting sites in αB-crystallin were identified by matrix-assisted laser desorption ionization-time-of-flight mass spectrometry (MALDI-TOF MS) and nanospray quadrupole time-of-flight mass spectrometry (QqTOF MS/MS).

**Results:**

We found that the βA3/A1_102–117_ peptide interacted with αB-crystallin regions ^70^LEKDR^74^, ^83^HFSPEELKVK^92^, ^91^VKVLGDVIEVHGK^103^, ^93^VLGDVIEVHGKHEER^107^, and ^121^KYR^123^, which are part of the α-crystallin domain, and were previously shown to be part of the functional chaperone site in αB-crystallin. The βA3/A1_102–117_ peptide also interacted with regions at the COOH-terminal extension of αB-crystallin, ^150^KQVSGPER^157^, ^164^EEKPAVTAAPK^174^, and ^164^EEKPAVTAAPKK^175^. When two of the hydrophobic residues of βA3/A1_102–117_ peptide were replaced with hydrophilic residues, the resulting substituted peptide, SDADHGERLMSFRPIC, did not show the anti-chaperone property.

**Conclusions:**

This study confirmed the interactions between a low molecular weight peptide derived from βA3/A1-crystallin found in aged and cataract lenses and αB-crystallin. The binding of βA3/A1_102–117_ peptide to the chaperone site and the COOH-terminal extension of αB-crystallin may explain its anti-chaperone property.

## Introduction

Human lens crystallins are organized into three classes, α-, β-, and γ-crystallins, based on sequence homology and the size of aggregates isolated under physiologic conditions. Since lens crystallins undergo little or no turnover, there is opportunity for numerous post-translational modifications as the lens ages [1-4]. Some modifications of lens crystallins identified in young clear lenses may reflect normal development and maturation of the lens [5,6] whereas other modifications associated with aged lenses- such as deamidation, phosphorylation, truncation, glycation, oxidation, and cross-linking could negatively impact crystallin conformation, aggregation state, or solubility, resulting in increased light scattering and eventual loss of transparency in aged lenses [7-10].

In comparison to normal lenses, cataract lenses show greater fragmentation of crystallins due to proteolysis [1,11-13]. Increased crystallin fragmentation has also been reported in aged and cataract lenses of non-human species [6,14-17]. It has been reported that proteolysis may be a contributing factor in the insolubilization of crystallins occurring during normal maturation of lens or during cataract formation in human and bovine lenses [18]. It has been shown that the fragments of βA3/A1- and βB1-crystallins are selectively insolubilized during cataract development compared to normal aging [13]. These authors also reported increased crystallin truncation, the deamidation of Asn to Asp residues, and the oxidation of a Trp residue in cataract lenses.

Several proteolytic enzymes have been shown to play a role in aging of the lens and cataract formation [19-25]. Activities of several peptidases were reported to be highest in the outer cortical fibers and decreased to one half or below in the inner cortical fibers and nucleus. An inverse correlation between peptidase activities and the amount of crystallin fragments was observed in different regions of the lens. The amount of crystallin fragments and the amount of water-insoluble proteins were greater in the lens nucleus than in the outer cortical fibers [21]. An age-dependent decline in proteasome activity and a concomitant accumulation of modified proteins in human lens has been reported earlier [26]. Increased opacification of the lens nucleus in cataract was significantly correlated with decreased peptidase activities of the proteasome [27]. Although a protein oxidation reaction may render the protein susceptible to proteolysis, heavily oxidized proteins appear to first aggregate (via new hydrophobic and ionic bonds) and then to form covalent cross-links that make them highly resistant to proteolysis by 20S proteasome [28]. In fact, these aggregated, cross-linked oxidized proteins actually bind to the 20S proteasome and inhibit its ability to degrade the oxidized forms of other proteins [29].

Low molecular weight crystallin fragments have been isolated and characterized from both the water-soluble and water-insoluble fractions of the lens with greater prevalence in the water-insoluble fractions [11,21,30-32]. As many as 13 crystallin fragments with molecular masses between 3 and 17 kDa and originating from α-, β-, and γ-crystallins have been isolated from the water-soluble, high molecular weight aggregates of 60-80-year-old human lenses [31]. Accumulation of such crystallin fragments may be a cause for age-related lens opacity. It has been hypothesized that the interaction of short peptides derived from crystallins with lens proteins may increase the formation of high molecular weight aggregates and the scattering of light [33]. Though it is hypothesized that cataract develops as a result of improper interaction of crystallin fragments generated by proteolysis [25], the underlying mechanism is not clear. Earlier work by our group has shown that in vitro-oxidized crystallin peptides enhance the aggregation of β_L_-crystallin and γ-crystallin and also exhibit anti-chaperone-like properties [33-35]. Interaction of peptide fragments with lens proteins and their aggregation-enhancing nature may have some implications for age-related cataract formation.

Recent studies in our laboratory have revealed the presence of 23 low molecular weight (<3.5 kDa) peptides in the urea-soluble fractions of normal young, normal aged, and aged cataract human lenses, and the amount of crystallin fragments was found to increase with age [36]. Out of these 23 peptides, 15 were derived from αA- and αB-crystallin. Most of the 15 came from the NH_2_-terminal region, a few from the α-crystallin domain, and none from the COOH-terminal extension. Two crystallin peptides predominant in the nucleus of aged lens and cataract lens but not in young lens, αB_1–18_ (MDIAIHHPWIRRPFFPFH, GenBank NP_001876) and βA3/A1_102–117_ (SD(N)AYHIERLMSFRPIC, GenBank NP_005199), increased the molecular mass, polydispersity, and hydrodynamic radius of αA- and αB-crystallins [36]. Our study also showed that the peptides, αB_1–18_ and βA3/A1_102–117_, increased the scattering of light by β- and γ-crystallins and alcohol dehydrogenase (ADH) and also reduced the chaperone-like activity of α-crystallin.

The present study was undertaken to identify the binding sites for the βA3/A1_102–117_ peptide in αB-crystallin. We used the photoactive cross-linker sulfo-SBED (sulfo-succinimidyl-2-[6-(biotinamido)-2-{p-azidobenzamido}-hexanoamido] ethyl-1–3 dithio propionate) [37] to confirm the interaction between αB-crystallin and the βA3/A1_102–117_ peptide. The βA3/A1_102–117_ peptide interacting sites in αB-crystallin are reported here.

## Methods

### Reagents

Trypsin gold (mass spectrometry grade) was purchased from Promega Corporation (Madison, WI). Sulfo-SBED, immobilized monomeric avidin kit, and micro-bicinchoninic acid (BCA) protein assay kit were procured from Pierce (Rockford, IL). Trypsin inhibitor, TLCK (N α-p-tosyl-L-lysine chloromethyl ketone), was obtained from Sigma Chemical Company (St. Louis, MO). The βA3/A1_102–117_ peptide, SDAYHIERLMSFRPIC, and the substituted peptide, SDADHGERLMSFRPIC (in which the tyrosine and isoleucine corresponding to 105 and 107 in βA3/A1-crystallin are replaced by aspartate and by glycine, respectively), were synthesized at the University of Missouri core facility and purified by HPLC. Their masses (1936.9 Da and 1833 Da, respectively) were ascertained by matrix-assisted laser desorption ionization-time-of-flight mass spectrometry (MALDI-TOF MS). Blotting grade avidin-horseradish peroxidase conjugate was obtained from Bio-Rad Laboratories (Hercules, CA). Bovine β_L_-crystallin was isolated from lens extract using the procedure described earlier [35]. All other chemicals were of analytical grade.

### Synthesis of recombinant human αB-crystallin

Human αB-crystallin cDNA (obtained from J.M. Petrash, Washington University, St. Louis, MO) was cloned into the pET-23d (+) vector (Novagen, Madison, WI), and the recombinant protein was expressed in *Escherichia coli* BL21(DE3) pLysS cells (Invitrogen, Carlsbad, CA) as described earlier [38]. The protein was purified by size exclusion chromatography followed by ion-exchange chromatography as described previously [39].

**Figure 1:**
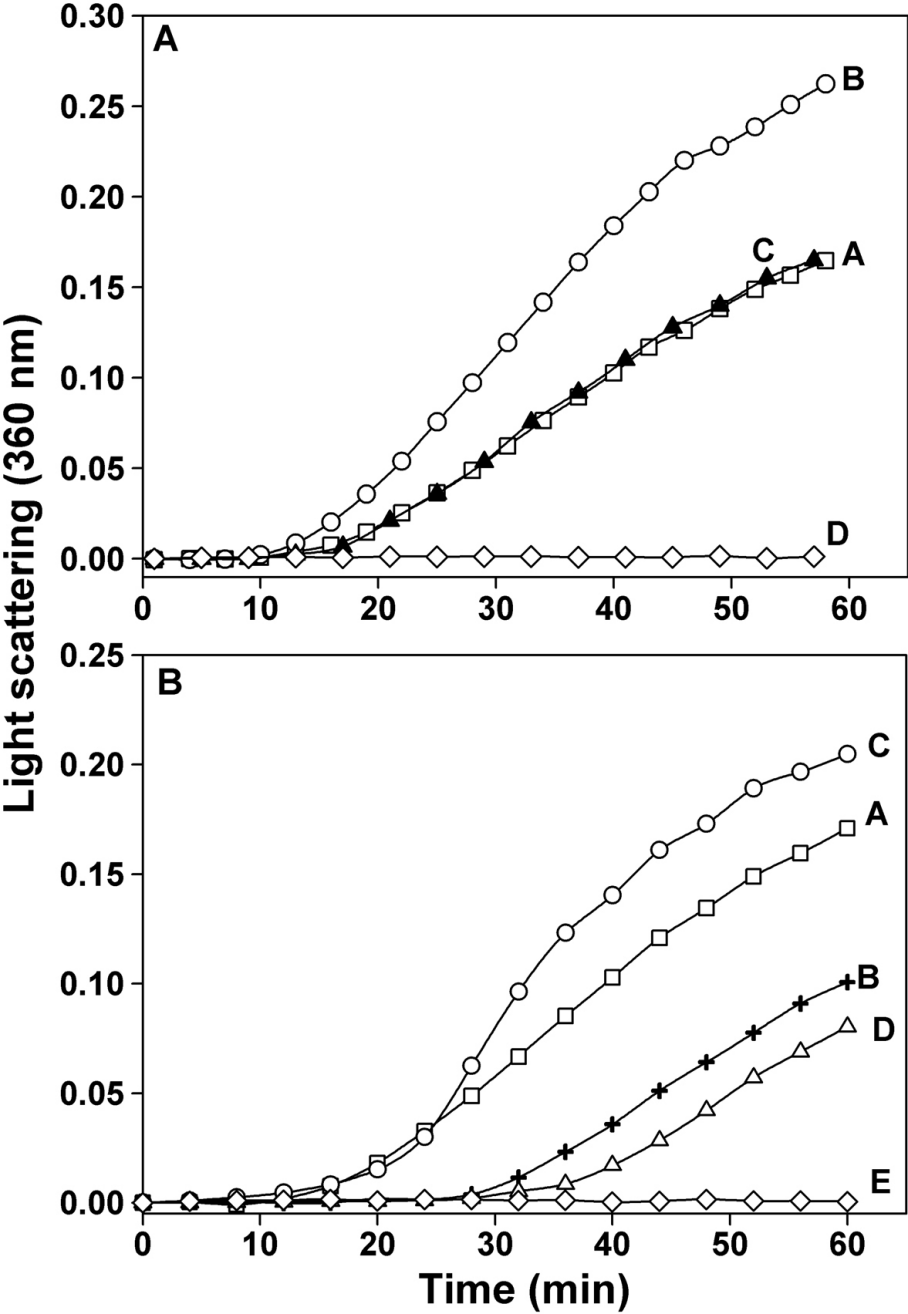
Aggregation-enhancing and anti-chaperone property of βA3/A1_102-117_ peptide. **A**: The effect of the βA3/A1_102–117_ peptide (SDAYHIERLMSFRPIC) and the substituted peptide (SDADHGERLMSFRPIC) on thermal aggregation of β_L_-crystallin shown. β_L_-crystallin (100 μg) was incubated at 55 °C with 60 μg of the peptides for 60 min. βA3/A1_102-117_ peptide enhanced the light scattering by denaturing β_L_-crystallin whereas the substituted peptide did not. A, β_L_-crystallin; B, β_L_-crystallin + βA3/A1_102–117_ peptide; C, β_L_-crystallin + substituted peptide; D, βA3/A1_102–117_ or substituted peptide alone. **B**: The effect of βA3/A1_102–117_ peptide (SDAYHIERLMSFRPIC) and the substituted peptide (SDADHGERLMSFRPIC) on the chaperone-like activity of αB-crystallin against denaturing β_L_-crystallin is illustrated. β_L_-crystallin (100 μg) was incubated at 55 °C in the presence of 5 μg of αB-crystallin with or without 60 μg of the peptides for 60 min. In the presence of βA3/A1_102-117_ peptide, the chaperone-like activity of αB-crystallin against denaturing β_L_-crystallin was lost. The substituted peptide however, did not decrease the chaperone-like activity of αB-crystallin. A, β_L_-crystallin; B, β_L_-crystallin + αB-crystallin; C, β_L_-crystallin + αB-crystallin + βA3/A1_102–117_ peptide; D, β_L_-crystallin + αB-crystallin + substituted peptide; E, αB-crystallin + βA3/A1_102–117_ or substituted peptide.

### Effect of βA3/A1_102–117_ peptide on the thermal aggregation of β_L_-crystallin and the chaperone function of αB-crystallin

The βA3/A1_102–117_ peptide (SDAYHIERLMSFRPIC) as well as the substituted peptide (SDADHGERLMSFRPIC) were dissolved in 50 μl of DMSO and diluted to 0.5 ml with 0.05 M phosphate buffer containing 0.15 M NaCl (pH 7.4), hereafter referred to as phosphate buffered saline (PBS). The peptide concentration was measured using the micro-BCA assay method. Thermal aggregation assay of bovine β_L_-crystallin and chaperone assay of αB-crystallin were performed according to the method previously described [35]. Briefly, substrate protein, β_L_-crystallin (100 μg), was heat-denatured at 55 °C in 1 ml of PBS for 60 min in the presence or absence of αB-crystallin (5 μg) and the βA3/A1_102–117_ peptide or the substituted peptide (60 μg). Aggregation was monitored by recording the light scattering at 360 nm as a function of time at 55 °C in a Shimadzu spectrophotometer equipped with a temperature-controlled multicell transporter.

**Figure 2:**
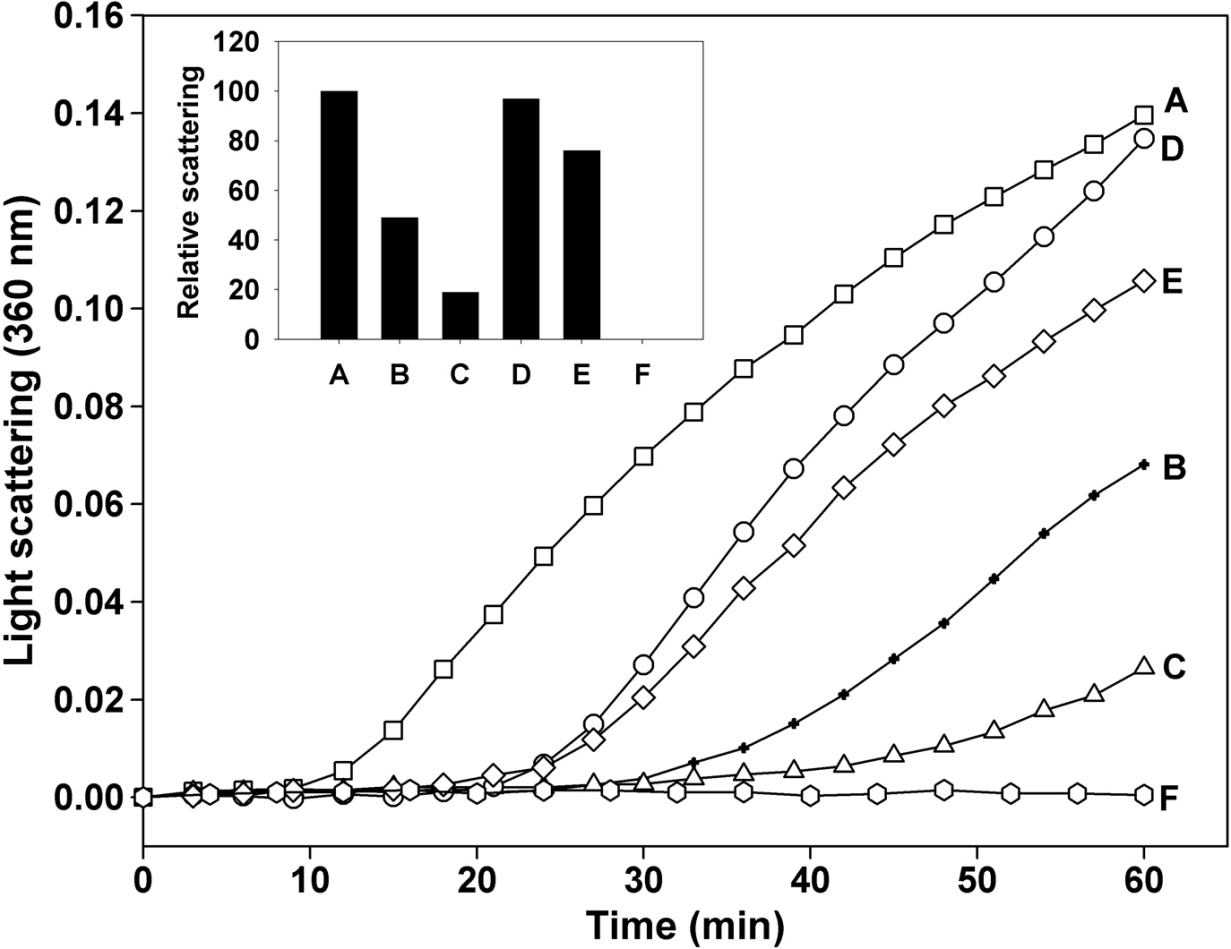
Chaperone-like activity of αB-crystallin-βA3/A1_102–117_ peptide complex against denaturing β_L_-crystallin. β_L_-crystallin (100 μg) was incubated at 55 °C in presence of 5 μg and 10 μg of αB-crystallin or αB-crystallin-βA3/A1_102–117_ peptide complex for 60 min and the light scattering was measured as described under methods. The results show that prior interaction of αB-crystallin with βA3/A1_102-117_ peptide diminished its chaperone-like activity against denaturing β_L_-crystallin. **A**, β_L_-crystallin; **B**, β_L_-crystallin + 5 μg αB-crystallin; **C**, β_L_-crystallin + 10 μg αB-crystallin; **D**, β_L_-crystallin + 5 μg αB-crystallin-βA3/A1_102–117_ peptide complex; **E**, β_L_-crystallin + 10 μg αB-crystallin-βA3/A1_102–117_ peptide complex; **F**, αB-crystallin or αB-crystallin-βA3/A1_102–117_ peptide complex alone. Insert: Relative light scattering by β_L_-crystallin in presence of αB-crystallin or αB-crystallin-βA3/A1_102–117_ peptide complex. Scattering by β_L_-crystallin alone at 60 min is considered to be 100%.

### Effect of pre-incubation with βA3/A1_102–117_ peptide on the chaperone function of αB-crystallin

To determine whether prior binding of the βA3/A1_102–117_ peptide alters the chaperone-like activity of αB-crystallin toward denaturing proteins, 50 μg of αB-crystallin was incubated with 100 μg of βA3/A1_102–117_ in a total volume of 0.2 ml of PBS at 37 °C for 12 h. A sample containing αB-crystallin alone served as a control. After the incubation, samples were briefly centrifuged to separate any visible precipitate. The supernatant was injected into a TSK G5000PW_XL_ (Tosoh Bioscience, Montgomeryville, PA) size exclusion column fitted to an HPLC system with an ultraviolet (UV) detector and equilibrated with PBS. The peak containing the αB-crystallin-peptide complex was pooled (free of unbound peptide) and used in the chaperone assay as described in the previous section.

**Figure 3:**
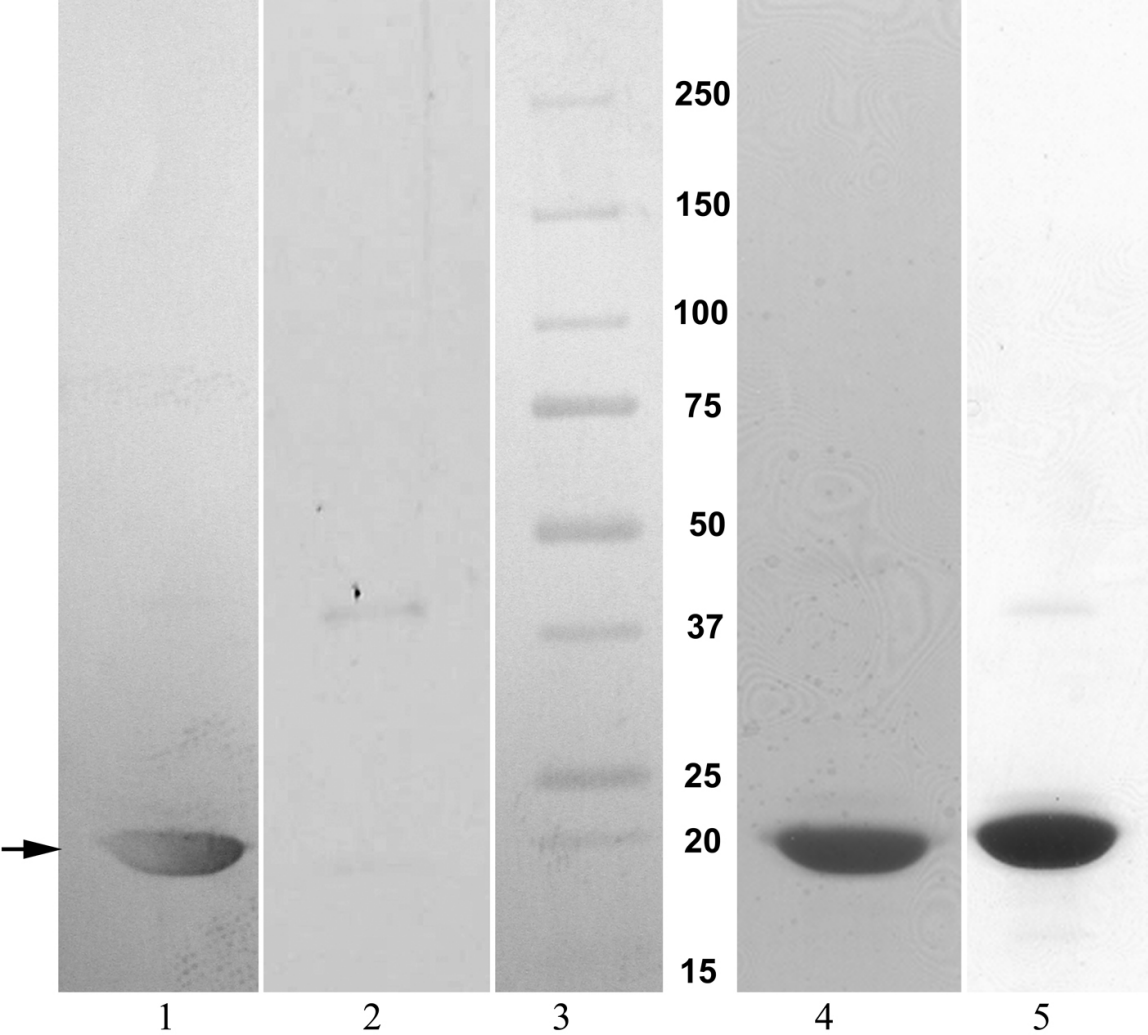
Western blot and SDS–PAGE of αB-crystallin treated with sulfo-SBED-derivatized βA3/A1_102–117_ peptide and substituted peptide. The blots were probed with avidin-horseradish peroxidase conjugate, and detection was done using a mixture of 4-chloronaphthol and hydrogen peroxide. Western blot lanes 1 and 2 correspond to SDS–PAGE of lanes 4 and 5 stained with coomassie blue. Lane 1- αB-crystallin interacted with sulfo-SBED-derivatized βA3/A1_102–117_ peptide; Lane 2- αB-crystallin interacted with sulfo-SBED-derivatized substituted peptide; Lane 3- Molecular weight markers; Lane 4- αB-crystallin interacted with sulfo-SBED-derivatized βA3/A1_102–117_ peptide; Lane 5- αB-crystallin interacted with sulfo-SBED-derivatized substituted peptide. The arrow indicates the position of αB-crystallin.

### Identification of βA3/A1_102–117_ peptide binding sites in αB-crystallin

To identify the βA3/A1_102–117_ peptide binding sites in αB-crystallin, the purified peptide was derivatized with sulfo-SBED, a biotin-containing and photoactivable as well as heterotrifunctional and amine-group specific reagent [37]. The procedure described by the supplier (Pierce) was used. In brief, 1.78 mg of βA3/A1 peptide was dissolved in 10 μl DMSO and the volume was made up to 50 μl with PBS. Then, 2.5 mg of sulfo-SBED dissolved in 10 μl DMSO and made up to 50 μl with PBS was added to the peptide, and the final volume of the reaction mixture was made up to 0.3 ml with phosphate buffer. This achieved a threefold molar excess of the sulfo-SBED over the peptide. The mixture was incubated in the dark at room temperature for 45 min. The unreacted sulfo-SBED was removed by dialyzing extensively against PBS using a 1000 Da cut-off membrane. Similarly, the substituted peptide, SDADHGERLMSFRPIC, was derivatized with sulfo-SBED for use in the interaction studies.

To study the interaction between the sulfo-SBED-derivatized βA3/A1_102–117_ peptide (or the substituted peptide) and αB-crystallin, 0.25 mg of the derivatized peptide was incubated with 0.25 mg of human recombinant αB-crystallin at 37 °C for 2 h. Subsequently, the mixture was filtered in a 10,000 Da cut-off centrifugal filter to remove unbound peptide (all steps were performed in the dark). Following this, the sample was photolyzed on an ice bath for 15 min using a UV lamp (365 nm) held at a distance of 2 cm from the sample. Transfer of the biotin label from the sulfo-SBED-derivatized peptide to αB-crystallin was confirmed by SDS–PAGE followed by western blot. The intensities of labeled bands in blots were quantified using Kodak 1D image analysis software (Eastman Kodak Company, Rochester, NY). The labeled αB-crystallin was then reduced by DTT and alkylated with iodoacetamide. Following this, the sample was dialyzed extensively for 24 h against 50 mM Tris (pH 7.6) containing 1 mM CaCl_2_ using a 15,000 Da cut-off membrane and digested by mass spectrometry grade trypsin (1:25 w/w) at 37 °C for 12 h. Trypsin digestion was terminated by the addition of the trypsin inhibitor, TLCK (1 mM); the sample was filtered through a microcon 10 kDa filter (Millipore, Bedford, MA), and the filtrate was collected. Trypsin-digested αB-crystallin peptides having the biotin group derived from the sulfo-SBED cross-linker were isolated by Pierce immobilized monomeric avidin affinity gel chromatography [37]. The biotin-labeled peptides were concentrated by Speed Vac, desalted by C-18 spin columns, and analyzed in an Applied Biosystems 4700 MALDI TOF/TOF mass spectrometer. The sequences of the labeled peptides were determined by nanospray quadrupole time-of-flight mass spectrometry (QqTOF MS/MS).

**Figure 4:**
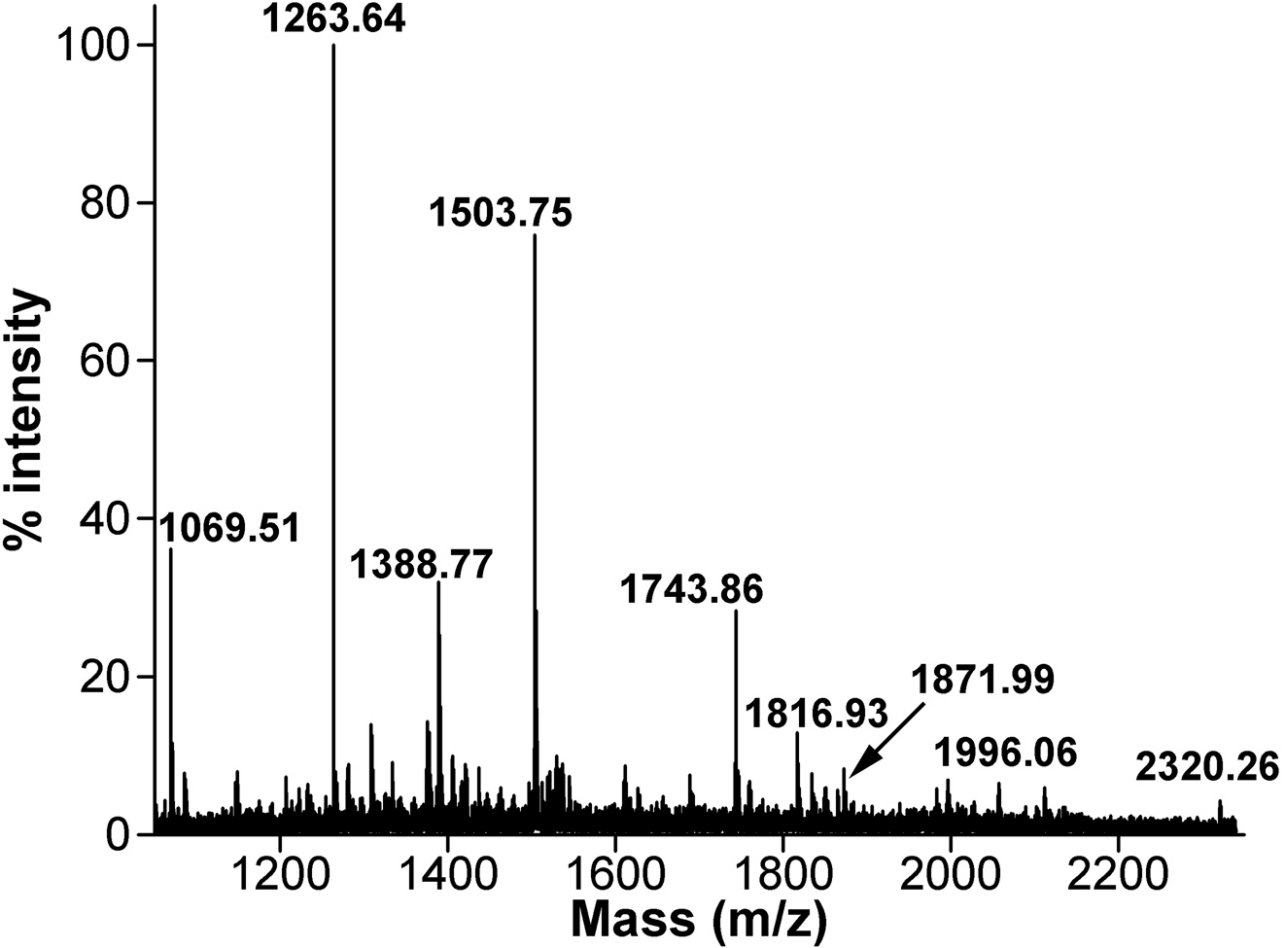
MALDI-TOF MS spectrum of biotin-labeled αB-crystallin tryptic peptides purified by avidin affinity chromatography. Sulfo-SBED-derivatized βA3/A1_102–117_ peptide was allowed to interact with αB-crystallin then was photolyzed and digested with trypsin as described under Methods before chromatography. Masses of the αB-crystallin tryptic peptides carrying the biotin label are indicated in the figure. The sequences of these peptides are shown in Figure 5.

## Results

### Effect of βA3/A1_102–117_ peptide on the thermal aggregation of bovine β_L_-crystallin and the chaperone-like action of αB-crystallin

The βA3/A1_102–117_ peptide found in aged lenses is present in both deamidated (at Asn_103_) and non-deamidated forms in vivo. Both the native peptide and the deamidated form of the same peptide displayed similar anti-chaperone activity (data not shown here). In this study, we used the deamidated form of the peptide. Thermal denaturation of β_L_-crystallin at 55 °C resulted in aggregation and light scattering. The addition of the βA3/A1_102–117_ peptide to the incubation mixture resulted in increased light scattering by β_L_-crystallin by 58% whereas the substituted peptide increased the light scattering only by 4% at 60 min. The βA3/A1_102–117_ peptide by itself did not scatter light (Figure 1A). When the β_L_-crystallin was heat-denatured in the presence of αB-crystallin, the light scattering was decreased by 41% at 60 min due to the chaperone-like action of αB-crystallin. However, when β_L_-crystallin was denatured in the presence of αB-crystallin and βA3/A1_102–117_ peptide, the light scattering increased by 20% showing the decreased chaperone action of αB-crystallin. The substituted peptide, on the other hand, caused a slight improvement in the chaperone action of αB-crystallin. The light scattering by denatured β_L_-crystallin was decreased by 53% in the presence of αB-crystallin and substituted peptide (Figure 1B). The αB-crystallin-βA3/A1_102–117_ peptide complex showed a lowered ability to prevent thermal aggregation of β_L_-crystallin. This complex (5 μg) decreased the light scattering by heat-denatured β_L_-crystallin at 60 min by only 3% (Figure 2, curve D) as opposed to a 51% decrease caused by an equivalent amount of the control, αB-crystallin (Figure 2, curve B). When a higher concentration of αB-crystallin-βA3/A1_102–117_ peptide complex (10 μg) was used in the assay, it caused only a 24% decrease in light scattering (Figure 2, curve E) compared to the 81% decrease caused by an equivalent amount of the control, αB-crystallin (Figure 2, curve C).

**Figure 5:**
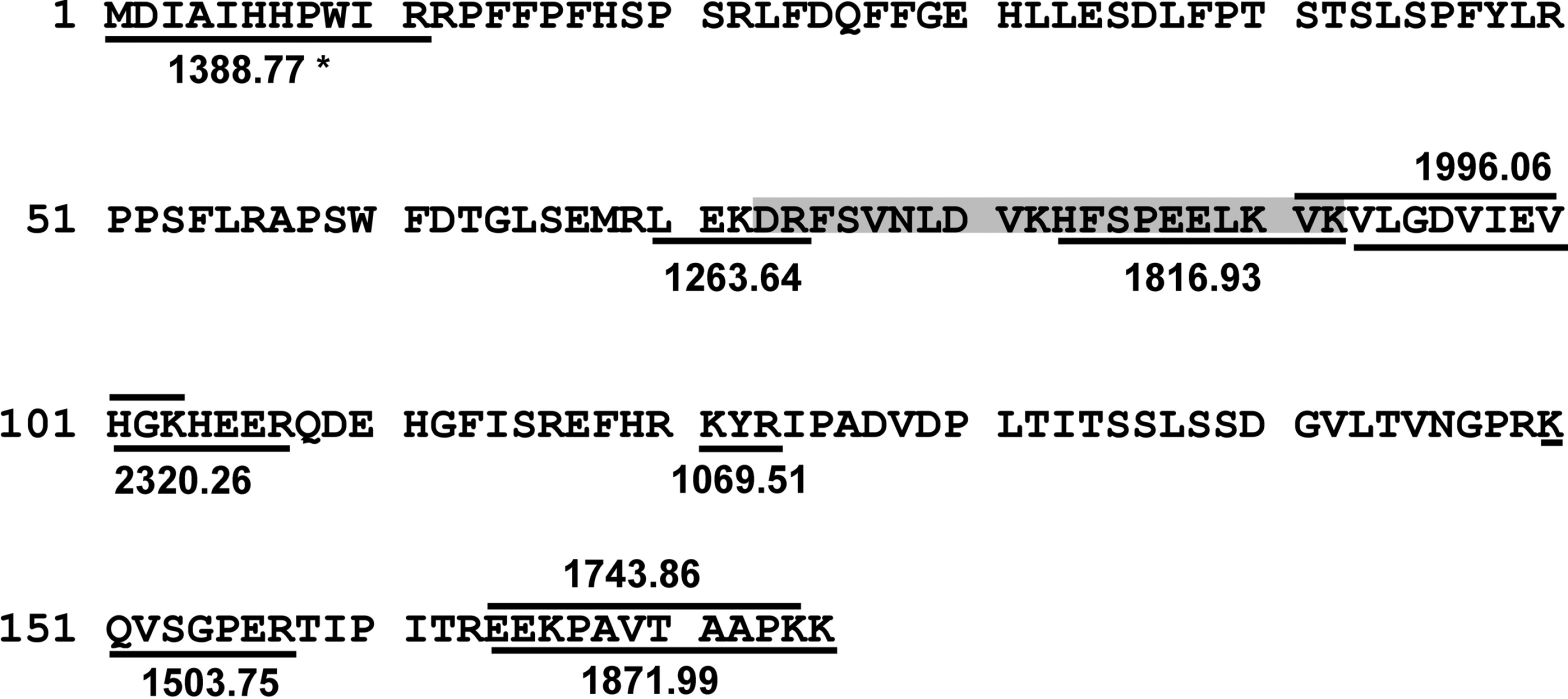
Amino acid sequence of αB-crystallin showing the βA3/A1_102–117_ peptide interacting sites (underlined). The chaperone region previously identified by us is shaded in gray. Biotin-labeled αB-crystallin peptides were analyzed by MALDI-TOF MS, and their sequences were assigned by nanospray QqTOF MS/MS. The masses of the biotin-labeled αB-crystallin peptides are shown adjacent to their sequences. *NH_2_-terminal peptide was consistently observed in the MALDI-TOF MS without a sulfo-SBED label. This peptide may be interacting with other labeled peptides and co-eluting during the monomeric avidin purification step.

### Identification of βA3/A1_102–117_ peptide binding sites in recombinant αB-crystallin

Photoinsertion of the biotin label from the derivatized βA3/A1_102–117_ peptide to αB-crystallin was confirmed by SDS–PAGE and western blot analysis (Figure 3, lane 1). The derivatized substituted peptide incorporated the biotin label into αB-crystallin to a significantly lesser extent than the native peptide (about 1.2% of the band intensity in western blot as compared to βA3/A1_102–117_ peptide) as seen in Figure 3, lane 2.

To identify the βA3/A1_102–117_ peptide interacting sites in αB-crystallin, the tryptic digest of αB-crystallin containing the biotin-labeled peptides was enriched by immobilized monomeric avidin affinity gel chromatography. The masses of the labeled peptides were determined by MALDI TOF/TOF MS. Since sulfo-SBED contains a cleavable disulfide bond, reduction and alkylation of the labeled αB-crystallin with iodoacetamide will add a net mass of 603.22 Da to the mass of the αB-crystallin-derived peptide containing the biotin label. Before assigning the amino acid sequence for each biotin-labeled αB-crystallin peptide, the presence of biotin label in those peptides was confirmed by detection of the fragment ions generating specifically from sulfo-SBED during MS/MS analysis [34]. MALDI-TOF MS spectra of biotin-labeled αB-crystallin peptides obtained from monomeric avidin affinity column is shown in Figure 4. The peptide peaks labeled in Figure 4 were analyzed further by nanospray QqTOF MS/MS. The amino acid sequence of those peptides is indicated in the αB-crystallin sequence in Figure 5. The amino acid sequences of tryptic digest peptides of αB-crystallin carrying the biotin label and their masses (in parentheses) were ^70^LEKDR^74^ (1263.64 Da), ^83^HFSPEELKVK^92^ (1816.93 Da), ^91^VKVLGDVIEVHGK^103^ (1996.06 Da), ^93^VLGDVIEVHGKHEER^107^ (2320.26 Da), ^121^KYR^123^ (1069.51 Da), ^150^KQVSGPER^157^ (1503.75 Da), ^164^EEKPAVTAAPK^174^ (1743.86 Da), and ^164^EEKPAVTAAPKK^175^ (1871.99 Da). A peptide with a mass of 1388.77 Da without any modifications, corresponding to residues 1–11 in αB-crystallin, was also found among the purified peptides.

## Discussion

The presence of crystallin fragments in water-soluble and water-insoluble fractions of lens proteins has been known for some years [11,40]. We first suggested that anti-chaperone peptides accumulating in vivo may be contributing to lens opacification during aging [33]. Recent studies in our laboratory have revealed the presence of 23 low molecular weight (<3.5 kDa) peptides in the urea-soluble fractions of normal young, normal aged, and aged cataract human lenses, and the amount of crystallin fragments was found to increase with age [36]. Our study also showed that βA3/A1_102–117_ peptide, found in aged and cataract lens, increased the scattering of light by β- and γ-crystallins and alcohol dehydrogenase (ADH). βA3/A1_102–117_ peptide also exhibited an anti-chaperone property by decreasing the ability of α-crystallin to prevent aggregation of β- and γ-crystallins and non-crystallin protein substrates, a process believed to be essential for maintenance of lens transparency [36].

The present study has confirmed the aggregation-enhancing property of the βA3/A1-crystallin-derived peptide, ^102^SDAYHIERLMSFRPIC^117^, since it promoted the aggregation of β_L_-crystallin during thermal denaturation (Figure 1A). The presence of this peptide in the assay mixture reduced the ability of αB-crystallin to protect β_L_-crystallin against thermal aggregation (Figure 1B). Since the βA3/A1_102–117_ peptide interacts with both β_L_-crystallin and αB-crystallin, the enhanced light scattering observed in its presence may be due to a combination of its interaction with the substrate as well as the chaperone protein. To confirm the anti-chaperone nature of the peptide, we pretreated αB-crystallin with βA3/A1_102–117_ peptide and separated the complex from the free peptide. The αB-crystallin-βA3/A1_102–117_ peptide complex showed a considerable loss of chaperone-like activity against heat-denatured β_L_-crystallin (Figure 2). Since free peptide was removed before the assay, we believe that the decreased chaperoning ability of αB-crystallin was specifically due to the binding of the peptide to αB-crystallin. When the βA3/A1_102–117_ peptide was derivatized with sulfo-SBED, incubated with αB-crystallin, and photolyzed, the biotin label transferred to αB-crystallin (Figure 3, lane 1). The MALDI-TOF MS of the peptides derived from the αB-crystallin by trypsin digestion showed the presence of biotin from sulfo-SBED in several peptides (Figure 4). The identity of those peptides was obtained by sequencing in a nanospray QqTOF mass spectrometer. The sites on αB-crystallin where the label was observed (Figure 5) included the sequences, ^70^LEKDR^74^ and ^83^HFSPEELKVK^92^. Earlier, we showed that ^73^DRFSVNLDVKHFSPEELKVK^92^ is one of the well characterized functional chaperone sites in αB-crystallin [41]. Therefore, we can conclude that the anti-chaperone peptide βA3/A1_102–117_ binds to αB-crystallin at the chaperone site. The binding of the βA3/A1_102–117_ peptide to this substrate-binding site in αB-crystallin may have diminished the ability of the protein to effectively chaperone the denaturing β_L_-crystallin.

Other sites of interaction between the βA3/A1_102–117_ peptide and αB-crystallin included the sequences, ^91^VKVLGDVIEVHGKHEER^107^ and ^121^KYR^123^. These sequences are part of the α-crystallin domain. Earlier, Arg_120_ was shown to be important for the chaperone-like activity of αB-crystallin [42]. A recent study demonstrated that ^73^DRFSVNLDVKHFS^85^ and ^101^HGKHEERQDE^110^ sequences in αB-crystallin inhibited the fibrillation of disease-related amyloidogenic proteins including α-synuclein and amyloid-β [43]. Other binding sites of the βA3/A1peptide to αB-crystallin included the COOH-terminal region of ^150^KQVSGPER^157^ and ^164^EEKPAVTAAPKK^175^. An earlier study has shown that mutations of the two terminal lysines (174 and 175) of αB-crystallin to leucine or glycine greatly reduced its chaperone-like activity. It was postulated that mutations in these two amino acids may have resulted in the COOH-terminus folding back on itself, losing its flexibility, and sterically hindering protein binding [44]. The COOH-terminal extensions of α-crystallins are considered polar, highly flexible, and solvent-exposed. These extensions also act as solubilizing agents for the relatively hydrophobic α-crystallin molecule and the high molecular weight complex that forms during the chaperone action [45].

αB-Crystallin with five amino acids deleted from its COOH-terminus was found in lenses of hereditary cataractous iCR/f rat, and this truncated crystallin showed decreased chaperone activity [46]. Protein pin array studies have identified ^157^RTIPITRE^164^ as one of the interactive sequences for chaperone activity in human αB-crystallin [47]. Deletion of the polar COOH-terminal sequence, ^155^PERTIPITREE^165^, from human αB-crystallin resulted in poor solubility and limited or no chaperone activity against unfolding β_L_-crystallin, alcohol dehydrogenase, and citrate synthase. These results demonstrated the importance of the COOH-terminal residues in the recognition, selection, and maintenance of solubility of unfolding substrate proteins [48]. As identified by protein pin-array studies, the ^150^KQVSGPER^157^, ^164^EEKPAVTAAPK^174^, and ^164^EEKPAVTAAPKK^175^ sequences, which are identified as peptide interacting sites in αB-crystallin in the present study, overlap with sequences involved in dimerization and assembly of human αB-crystallin [49]. The binding of the βA3/A1_102–117_ peptide to the COOH-terminal extension of αB-crystallin in the present study may have interfered with substrate binding and decreased the chaperoning ability of the protein. Our recent studies showed that the βA3/A1_102–117_ peptide caused partial precipitation of αB-crystallin [36]. We believe that the peptide interaction at the COOH-terminal extension of αB-crystallin may have led to decreased flexibility and decreased solubilization potential of αB-crystallin. Additionally, the interactions between crystallins and low molecular weight peptides may limit the access of these peptides to peptidases, resulting in the accumulation of peptides with aging.

The substituted peptide we used in this study, SDADHGERLMSFRPIC, had two hydrophobic residues (tyrosine and isoleucine) replaced by hydrophilic (aspartate and glycine) residues. The substitution abolished the ability of the peptide to induce β_L_-crystallin aggregation (Figure 1A) and to act as anti-chaperone (Figure 1B). This suggests that the hydrophobic amino acids play a critical role in the interaction of the anti-chaperone peptide with αB-crystallin. The substituted peptide can also be considered a control peptide for the label transfer study. If there were any non-specific interactions between the derivatized substituted peptide and αB-crystallin, we would have seen a transfer of biotin to αB-crystallin following photolysis. The fact that the αB-crystallin incubated with derivatized substituted peptide showed only about 1.2% of the label transfer when compared to the derivatized βA3/A1_102–117_ peptide during the blotting experiments (Figure 3 lane 2) suggests that the non-specific interaction and label transfer was insignificant in this study.

In summary, this study confirmed the interactions between βA3/A1_102–117_ peptide and αB-crystallin. The binding of this peptide to regions critical for chaperone action of αB-crystallin may explain its anti-chaperone property. The modulation of structural and functional properties of crystallins by low molecular weight peptides may play an important role in protein aggregation and cataract formation.
